# Expression Profiles of Circular RNA in Aortic Vascular Tissues of Spontaneously Hypertensive Rats

**DOI:** 10.3389/fcvm.2021.814402

**Published:** 2021-12-20

**Authors:** Ying Liu, Ying Dong, Zhaojie Dong, Jiawei Song, Zhenzhou Zhang, Lirong Liang, Xiaoyan Liu, Lanlan Sun, Xueting Li, Miwen Zhang, Yihang Chen, Ran Miao, Jiuchang Zhong

**Affiliations:** ^1^Heart Center and Beijing Key Laboratory of Hypertension, Beijing Chaoyang Hospital, Capital Medical University, Beijing, China; ^2^Department of Cardiology, Beijing Chaoyang Hospital, Capital Medical University, Beijing, China; ^3^Department of Respiratory and Critical Care Medicine, Beijing Institute of Respiratory Medicine and Beijing Chaoyang Hospital, Capital Medical University, Beijing, China; ^4^Department of Echocardiography, Beijing Chaoyang Hospital, Capital Medical University, Beijing, China

**Keywords:** competing endogenous RNAs network, microRNA, circular RNA, hypertensive vascular injury, ischemia heart disease

## Abstract

**Background:** Circular RNAs (circRNAs), as a kind of endogenous non-coding RNA, have been implicated in ischemic heart diseases and vascular diseases. Based on theirs high stability with a closed loop structure, circRNAs function as a sponge and bind specific miRNAs to exert inhibitory effects in heart and vasculature, thereby regulating their target gene and protein expression, *via* competitive endogenous RNA (ceRNA) mechanism. However, the exact roles and underlying mechanisms of circRNAs in hypertension and related cardiovascular diseases remain largely unknown.

**Methods and Results:** High-throughput RNA sequencing (RNA-seq) was used to analyze the differentially expressed (DE) circRNAs in aortic vascular tissues of spontaneously hypertensive rats (SHR). Compared with the Wistar-Kyoto (WKY) rats, there were marked increases in the levels of systolic blood pressure, diastolic blood pressure and mean blood pressure in SHR under awake conditions *via* the tail-cuff methodology. Totally, compared with WKY rats, 485 DE circRNAs were found in aortic vascular tissues of SHR with 279 up-regulated circRNAs and 206 down-regulated circRNAs. Furthermore, circRNA-target microRNAs (miRNAs) and the target messenger RNAs (mRNAs) of miRNAs were predicted by the miRanda and Targetscan softwares, respectively. Additionally, real-time RT-PCR analysis verified that downregulation of rno_circRNA_0009197, and upregulation of rno_circRNA_0005818, rno_circRNA_0005304, rno_circRNA_0005506, and rno_circRNA_0009301 were observed in aorta of SHR when compared with that of WKY rats. Then, the potential ceRNA regulatory mechanism was constructed *via* integrating 5 validated circRNAs, 31 predicted miRNAs, and 266 target mRNAs. More importantly, three hub genes (NOTCH1, FOXO3, and STAT3) were recognized according to PPI network and three promising circRNA-miRNA-mRNA regulatory axes were found in hypertensive rat aorta, including rno_circRNA_0005818/miR-615/NOTCH1, rno_circRNA_0009197/ miR-509-5p/FOXO3, and rno_circRNA_0005818/miR-10b-5p/STAT3, respectively.

**Conclusions:** Our results demonstrated for the first time that circRNAs are expressed aberrantly in aortic vascular tissues of hypertensive rats and may serve as a sponge linking with relevant miRNAs participating in pathogenesis of hypertension and related ischemic heart diseases *via* the circRNA-miRNA-mRNA ceRNAnetwork mechanism.

## Introduction

Hypertension is a leading risk factor of ischemic heart diseases and hypertensive heart diseases that mainly leads to the high morbidity and mortality (1, 2). It is estimated that ~1.56 billion adults worldwide expected to be influenced by hypertension by 2025 (3). To our knowledge, cardiovascular damage is an essential pathological characteristic of hypertension. Especially, impaired vascular remodeling and endothelial dysfunction result in the evolution of coronary artery abnormalities (4), eventually causing ischemia heart disease. A variety of mechanisms lead to the generation of myocardial ischemia during hypertension (5). However, current studies on the effective biomarkers of hypertensive vascular injury are still limited.

Non-coding RNAs (ncRNAs) play critical roles in hypertension and hypertensive vascular disorders, including ribosomal RNAs, transfer RNAs, small nuclear RNAs, microRNAs (miRNAs), and long non-coding RNAs (lncRNAs) (6). Recently, circRNA is a kind of bioactive RNA molecules with a closed loop structure (7). The majority of circRNAs are widely available in cardiovascular system and have multiple critical specific functions in vascular physiology and homeostasis at the posttranscriptional level (8). Based on the high stability, circRNAs function as a sponge and bind specific miRNAs to exert inhibitory effects on regulating the gene and protein expression, *via* ceRNA mechanism (9). Several circRNAs have been involved in vascular injury in pulmonary arterial hypertension (9, 10). However, the regulatory roles and underlying mechanisms of circRNAs in hypertension and hypertensive vascular diseases remain largely unclear.

In current study, the differentially expressed (DE) circRNAs were detected in aortic vascular tissues of the spontaneously hypertensive rat (SHR) and the Wistar-Kyoto (WKY) rats by high-throughput RNA sequencing (RNA-Seq) analysis and the real-time polymerase chain reaction (RT-PCR) validation, respectively. More importantly, three promising circRNA-miRNA-mRNA regulatory axes network were highlighted in hypertensive rat aorta, providing potential therapeutic targets for hypertension and hypertensive vascular disorders.

## Materials and Methods

### Experimental Animals, Blood Pressure Measurement, and Tissue Preparation

A total of five male 13-week-old SHR and five age- and weight-matched WKY rats regarded as control groups were purchased from Beijing Vital River Laboratory Animal Technology Co., Ltd. All the experiments were conformed to the guidelines of animal experiments reported at Capital Medical University. Rats were maintained in a temperature and humidity-controlled animal room with 12:12 h day:night cycle, meanwhile different cages were used for each group. All of the animals were accessed to tap water and laboratory feed. Systolic blood pressure (SBP), diastolic blood pressure (DBP), and mean blood pressure (MBP) were non-invasively measured under awake conditions *via* tail-cuff methodology (Softron, BP-98A, Japan). The thoracic aorta was extracted, removed fat and connective tissues and frozen in liquid nitrogen immediately. Samples were shipped to the laboratory and stored at −80°C until analysis. All experiments were approved and performed in accordance with the National Institutes of Health guide for the care and use of Laboratory animals (NIH Publications No. 8023), Animal Research Ethics Committee of Beijing Chaoyang Hospital affiliated to Capital Medical University.

### The Elastica Van Gieson Staining

The EVG staining was intended for use in the histological demonstration of elastin in tissues and collagen fiber. The demonstration of elastic in tissue was useful in vascular diseases. The deparaffinize sections were hydrated in distilled water. Put the slides in Elastic stain solution for 15 min and washed the slide by use of running tap water. Then, the slides were kept in Sodium Thiosulfate Solution for 1 min and in Van Gieson's Solution for 2–5 min, and then rinse in two changes of 95% alcohol and dehydrate in absolute alcohol (Abcam, Cambridge, MA).

### Total RNA Isolation and Library Preparation for circRNA Sequencing

Total RNAs were isolated from thoracic aorta from SHR and WKY rats, a total amount of 5 μg RNA per sample was used as input material for the RNA sample preparations using TRIzol reagent (Invitrogen, USA). Furthermore, RNA purity was checked using the NanoPhotometer®spectrophotometer (IMPLEN, CA, USA) and RNA integrity was evaluated using the RNA Nano 6000 Assay Kit of the Bioanalyzer 2100 system (Aglient Technologies, CA, USA). Additionally, ribosomal RNA was depleted from total RNA using the Epicenter Ribozero™ rRNA Removal Kit (Epicenter, USA), and rRNA free residue was cleaned up by ethanol precipitation. Subsequently, the linear RNA was digested with 3U of RNase R (Epicenter, USA) per μg of RNA. The sequencing libraries were generated by NEBNext®Ultra™ Directional RNA Library Prep Kit for Illumina® (NEB, USA) following manufacturer's recommendations by Shanghai Genechem Co., Ltd., Shanghai, China. The clustering of the index-coded samples was performed on a cBot Cluster Generation System using TruSeq PE Cluster Kit v3-cBot-HS (Illumina) according to the manufacturer's instructions. After cluster generation, the libraries were sequenced on an Illumina platform and 150 bp paired-end reads were generated.

### Quality Control and circRNA Identification

The raw data of quality control was firstly processed through in-house perlscripts. After removing reads containing adapter, poly-N or low-quality reads, clean reads were left. Meanwhile, Q20, Q30, and GC content of the clean reads were calculated (Supplementary Table 1). Importantly, clean reads with high quality were applied among all the downstream analyses. Index of the reference genome was built using bowtie2 v2.2.8 and paired-end clean reads were aligned to the reference genome using Bowtie software (11).

### Differential Expression Analysis of circRNAs

Differential expression analysis between the two groups was assessed using the DESeq R package (1.10.1). DESeq provided statistical routines for determining differential expression in digital gene expression data using a model based on the negative binomial distribution. The resulting *P*-values were corrected using the Benjamini and Hochberg's approach for controlling the false discovery rate, shown as q values. Genes with an adjusted *P*-value or *q*-value < 0.05 were regarded as statistical significance.

### GO and KEGG Pathway of circRNAs and mRNAs in the Network

Gene Ontology (GO) (http://www.geneontology.org) and Kyoto Encyclopedia of Genes and Genomes (KEGG) signal pathway enrichment analysis (http://www.genome.jp/kegg) were conducted to explore the biological function of targeted genes. Gene function was categorized into three separate subgroups: biological processes (BPs), cellular components (CCs), and molecular functions (MFs), respectively.

### Validation of RNA Sequencing With Real Time Reverse Transcription-Polymerase Chain Reaction Analysis

Five pairs of SHR and WKY rats were employed to confirm the expression levels of DE circRNAs by RT-PCR analysis. In brief, total RNAs were reversed transcribed into cDNA using PrimeScript™ reverse transcription reagent kit (Takara Bio, Inc., Otsu, Japan) according to the manufacturer's protocol. Then, cDNAs were used for RT-PCR analysis to examine the expression of circRNA by the ABI Prism 7500 sequence detection system (Applied, Biosystems, CA). GADPH was served as a normalizing standard for all target circRNAs. The primers for each circRNAs were summarized in Supplementary Table 2. All samples were run in triplicate. The relative expression of selected circRNAs were calculated using the 2^−Δ*ΔCT*^ method.

### Prediction of circRNA-miRNA-mRNA Interaction Network and Identification of Hub Genes

We next predicted the sequence identity of the circRNA in human and rat by using Basic Local Alignment Search Tool (https://blast.ncbi.nlm.nih.gov/Blast.cgi) and the circAtlas 2.0 software (http://circatlas.biols.ac.cn/). Moreover, the altered circRNAs in hypertensive rat aorta with corresponding miRNAs and the potential target mRNAs were used to establish a circRNA-miRNA-mRNA interaction network. The miRNA-mRNA interactions were firstly predicted using Targetscan (www.targetscan.org/). The circRNA-miRNA-mRNA network to further visualize the interactions using Cytoscape 3.8.2 software. Moreover, the STRING database (http://string-db.org/) was used to establish the protein-protein interaction (PPI) of the predicted mRNA, and then visualized by using Cytoscape version 3.8.2 software. Subsequently, hub genes were also determined using cytoHubba app of Cytoscape.

### Statistical Analysis

The data analyses were conducted using SPSS software version 21.0 (IBM SPSS, Chicago, IL, USA). GraphPad Prism version 7.0 (GraphPad software, Inc., LaJolla, CA, USA) and Cytoscape version 3.8.2 were applied to generate the figures. All the data were presented as means ± standard deviations (SDs) and compared using the Student's *t*-test, and a *P*-value or *q-*value < 0.05 was regarded as significant. All experiments were carried out three times.

## Results

### Expression Profiling of Aortic circRNAs Between SHR and WKY Rats

**Figure 1:**
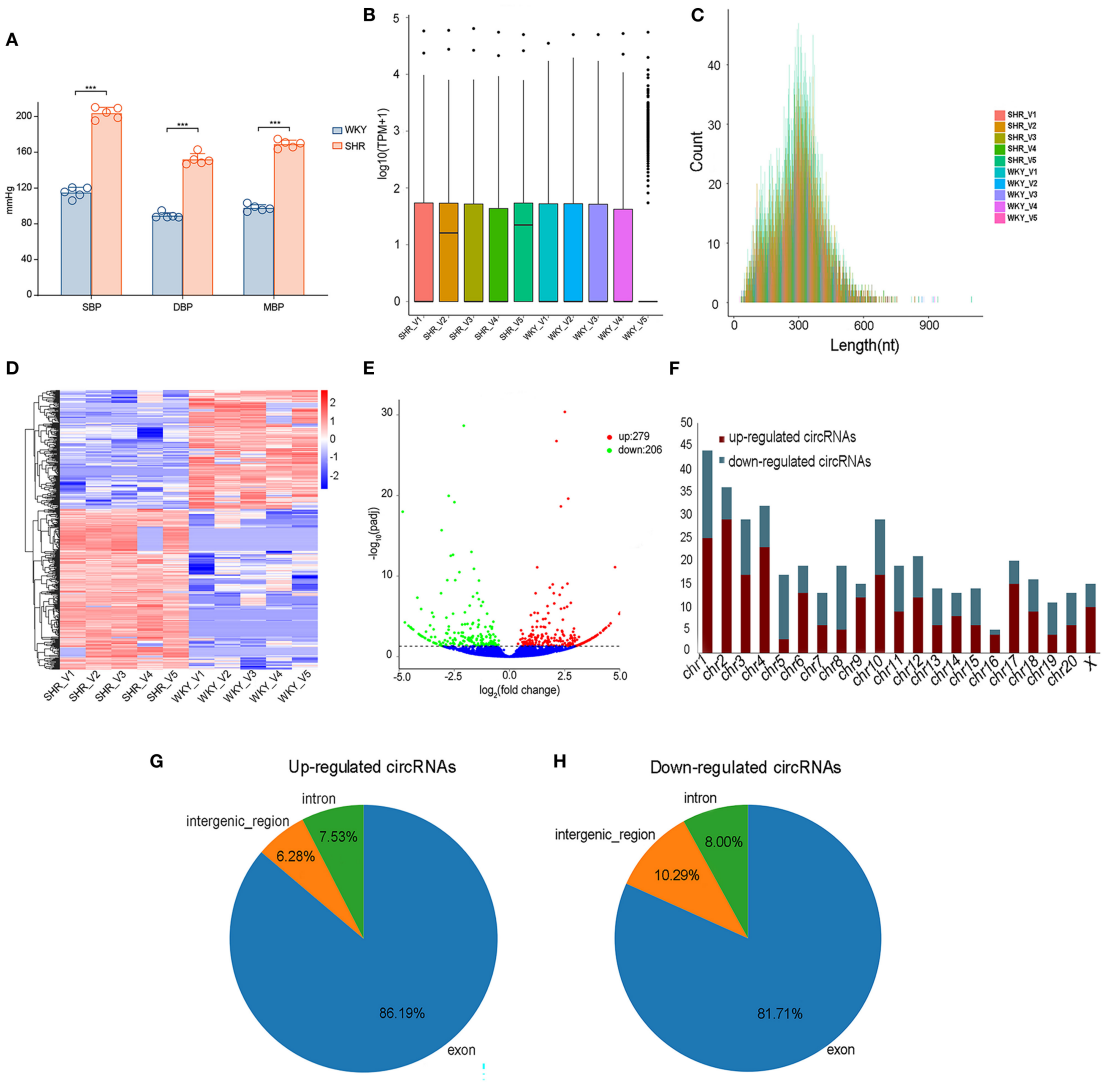
The expression profiling of vascular circRNAs was screened and analyzed by RNA-seq of aortic vascular tissues between SHR and WKY rats. **(A)** Compared with WKY controls, SBP levels, DBP levels and MBP levels were significantly elevated in SHR rats. **(B)** The data standardization of the total expression of the circRNAs. **(C)** The length of circRNA profiles. **(D)** The hierarchical cluster analysis was performed with the significantly different circRNA expression profiles in SHR and WKY aorta (Fold Change > 2 and *P* < 0.05); Red and blue denoted high and low relative expression, respectively, each circRNA was expressed by a single row of colored boxes and each sample was represented by a single column. **(E)** Volcano plot showed that the differential expression analysis of circRNAs (red, up-regulated; green, down-regulated). **(F)** The distribution of chromosomes of upregulated and downregulated circRNAs, respectively. **(G,H)** The identification of exon, intron and intergenic region of upregulated and downregulated circRNAs. RNA-seq, high-throughput RNA sequencing; WKY, Wistar-Kyoto; SHR spontaneously hypertensive rat; SBP, systolic blood pressure; DBP, diastolic blood pressure; MBP, mean blood pressure; circRNAs, circular RNAs.

Vascular circRNAs expressions between SHR and WKY rats were measured using RNA sequencing analysis. Firstly, compared with WKY rats, the levels of SBP, DBP and MBP were significantly elevated in SHR (Figure 1A). Furthermore, information of circRNAs was exhibited, including the raw reads, read counts, raw bases, GC content (%), and Q30% etc (Supplementary Table 1). The basic information and classifications of sequencing quality for circRNAs were revealed in aortic vascular tissues of SHR and WKY rats (Supplementary Figure 1). Box plots showing the circRNA profiles revealed the similar distributions of all datasets in the detected samples (Figure 1B). The circRNA transcripts were mostly 300 bp in length (Figure 1C). In addition, clustered heatmap was conducted to aortic circRNA according to their expression levels among rats (Figure 1D), indicating that the circRNA profiles were different between SHR and WKY rats. To determine these different circRNAs expressions in hypertension, the variation between each group was analyzed by the volcano plot. Significantly DE circRNAs were identified as *q* < 0.05 and |log2(fold changes) |>1. Totally, 485 DE circRNAs were detected in SHR (279 up-regulated and 206 down-regulated circRNAs) compared with WKY rats (Figure 1E). We further classified the distributions of DE circRNAs according to the chromosomes transcribed from, which demonstrated that among these DE circRNAs, most up-regulated circRNAs mainly came from chromosomes (chr) 1 (chr1), chr2, chr3, chr4, and chr10, whereas the down-regulated circRNAs came from chr1, chr3, chr5, chr8, and chr10 (Figure 1F). Moreover, among these DE circRNAs, abundant RNAs were generated most commonly from exons of protein-coding genes (86.19 and 81.71% from up-regulated and down-regulated circRNAs, respectively) (Figures 1G,H).

### GO and KEGG Pathway of DE circRNAs in Hypertensive Rat Aorta

**Figure 2:**
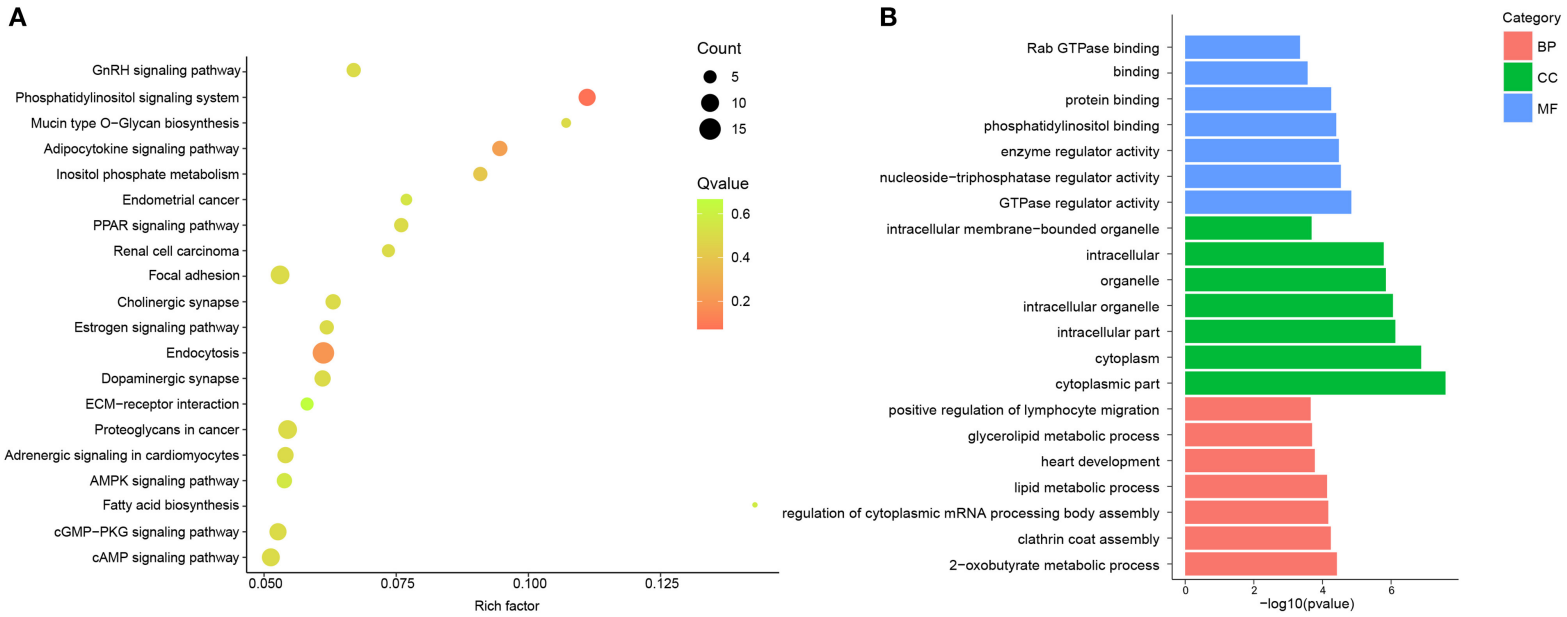
GO and KEGG pathway analysis of DE circRNAs between and SHR and WKY rat aorta. **(A)** Top 20 KEGG pathways for DE circRNAs were identified in SHR and WKY rat aorta. **(B)** Top 7 GO enriched terms were analyzed for biological process (BP), cellular component (CC), and molecular function (MF), respectively. GO, gene ontology; KEGG, Kyoto encyclopedia of genes and genomes; DE, differentially expressed; circRNAs, circular RNAs; WKY, Wistar-Kyoto; SHR spontaneously hypertensive rat.

To describe and synthesize potential mechanisms of confirmed circRNAs, the GO and KEGG pathways were analyzed in hypertensive rat aorta. The enriched pathways analyzed by KEGG were Endocytosis, Focal adhesion, cyclic adenosine monophosphate (cAMP) signaling pathway, AMP-activated protein kinase (AMPK), phosphatidylinositol (PI) signaling pathway and extracellular matrix (ECM)-receptor interaction (Figure 2A; Supplementary Table 3). In addition, GO enrichment analysis reflected the top 30 enriched GO term of the DE circRNAs (Supplementary Table 4). In the term of MF, the DE circRNAs were mainly enriched in Guanosine-Triphosphate hydrolase (GTPase) regulator activity. In the CC, the main term of GO analysis was cytoplasmic part. Besides, in the term of BP, the central term of was 2-oxobutyrate metabolic process (Figure 2B). Thus, the cAMP, AMPK, PI, and GTPase signaling pathways may be partially responsible for circRNAs- mediated vascular pathophysiology and homeostasis of hypertension.

### Confirmation of DE circRNAs Candidates by RT-PCR Between SHR and WKY Rats

**Figure 3:**
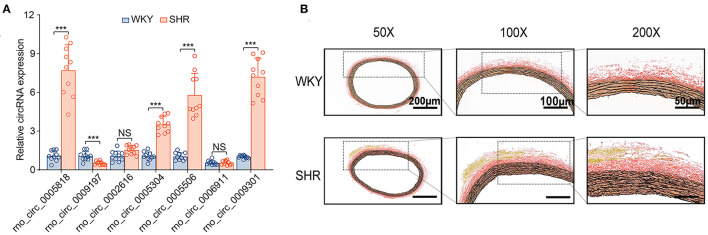
DE circRNAs candidates were validated by RT-PCR between SHR and WKY rat aorta. **(A)** The seven DE circRNAs were detected by the RT-PCR between SHR and WKY rat aorta. Five significant aortic circRNAs were confirmed in hypertensive rats, including rno_circRNA_0005818, rno_circRNA_0005304, rno_circRNA_0005506, rno_circRNA_0009301, and rno_circRNA_0009197. **(B)** The EVG staining illustrated that the regulation of circRNAs were associated with thin elastic fiber and thick collagen fiber. Black color represents elastic fiber, red color represents collagen fiber. *n* = 10 for each group, ***P < 0.001. DE, differentially expressed; circRNAs, circular RNAs; RT-PCR, real-time polymerase chain reaction; WKY, Wistar-Kyoto; SHR spontaneously hypertensive rat.

Subsequently, the top 20 significantly up-regulated and down-regulated circRNAs (*q* < 0.05 and | log2(foldchange)|>4) were listed in Table 1. Of which, seven DE circRNAs were chosen for further confirmation by RT-PCR analysis. The RT-PCR validation demonstrated the similar trends with RNA-seq, suggesting the reliability of our circRNAs expression profiles. Particularly, five significant circRNAs including rno_circRNA_0005818, rno_circRNA_ 0005304, rno_circRNA_0005506, rno_circRNA_0009301, and rno_circRNA_0009197 were validated by RT-PCR analysis (Figure 3A). The results indicated that the expressions of rno_circRNA_0005818, rno_circRNA_0005304, rno_circRNA_0005506, and rno_circ RNA_0009301 were significantly increased in SHR aorta (7.92-fold, *P* < 0.001; 3.42-fold, *P* < 0.001; 5.09-fold, *P* < 0.001; and 7.29-fold, *P* < 0.001, respectively). In addition, rno_circRNA_ 0009197 was significantly decreased in SHR aorta (2.26-fold, *P* < 0.001). More importantly, we found that there were highly similar homologous sequences in rno_circRNA_0009197 and rno_circRNA_0005506 with human circRNA, according to the criterion of evalue and identity by using Basic Local Alignment Search Tool and the circAtlas 2.0 software, respectively. Generally, the higher the similarity between sequences of human and rat circRNAs, the higher the possibility that they are homologous sequences. In addition, when the evalue is close to zero or zero, it is exactly a match sequences of human and rat circRNAs. Notably, rno_circ_0009197 and rno_circ_0005506 were identified highly homologous with hsa_circ_0018685| chr10:73337670-73559386 (identity 0.907; evalue 1.87E-18) and hsa_circ_0112669| chr1:237865277-237921076 (identity0.874; evalue 1.66E-105), respectively. Moreover, EVG staining showed aggravated elastin and collagen fiber of thoracic aorta in SHR compared with the WKY rats, characterized with thickened collagen fibers and augmented ECM deposition (Figure 3B), indicating that upregulations of rno_circRNA_0005818, rno_circRNA_0005304, rno_circRNA_0005506, and rno_circ_RNA_0009301 and downregulation of rno_circRNA_0009197 may play potential roles in aortic hypertrophy and remodeling of hypertensive rats.

**Table 1:** Top 20 significantly up-regulated and down-regulated circRNAs in SHR rat (|log2FoldChange| > 4, *Q*-value < 0.05).

**CircRNA**	***Q*-value**	**log2FoldChange**	**Regulation**	**chrom**	**strand**	**CircRNA_type**	**GeneSymbol**
rno_circRNA_0009301	1.51E-17	7.3709	Up	chr20	+	Exonic	–
rno_circRNA_0005304	2.28E-12	6.4932	Up	chr17	+	Intergenic	–
rno_circRNA_0008384	3.59E-09	5.9610	Up	chr1	–	Intergenic	–
rno_circRNA_0001706	8.23E-09	5.7243	Up	chr11	–	Exonic	Adcy5
rno_circRNA_0000407	3.37E-06	5.0298	Up	chr10	–	Exonic	Snx29
rno_circRNA_0010188	3.18E-06	5.0262	Up	chr2	+	Exonic	Frrs1
rno_circRNA_0000489	3.56E-06	5.0107	Up	chr10	+	Exonic	Sap30l
rno_circRNA_0012471	4.90E-06	4.9696	Up	chr4	+	Exonic	RGD1565355
rno_circRNA_0010453	7.82E-12	4.7779	Up	chr2	+	Exonic	Ddah1
rno_circRNA_0012704	7.38E-05	4.5727	Up	chr4	–	Intergenic	–
rno_circRNA_0002616	1.42E-04	4.4966	Up	chr12	–	Exonic	Fry
rno_circRNA_0005818	2.48E-04	4.3747	Up	chr17	–	Exonic	Dnajc1
rno_circRNA_0009971	3.39E-04	4.3142	Up	chr2	+	Exonic	Arnt
rno_circRNA_0018260	4.52E-04	4.2612	Up	chr9	–	Exonic	Smap1
rno_circRNA_0004376	5.72E-04	4.2419	Up	chr15	+	Exonic	Scara5
rno_circRNA_0005506	1.10E-03	4.1191	Up	chr17	–	Exonic	Ryr2
rno_circRNA_0011085	1.09E-03	4.1051	Up	chr3	–	Exonic	Fbn1
rno_circRNA_0011717	1.89E-03	3.9862	Up	chr3	–	Exonic	Golga1
rno_circRNA_0018702	2.53E-03	3.9256	Up	chr9	–	Exonic	Bard1
rno_circRNA_0004164	2.48E-03	3.921	Up	chr15	+	Exonic	Samd4a
rno_circRNA_0018394	1.70E-15	7.0728	Down	chr9	+	Exonic	Bivm
rno_circRNA_0018389	1.91E-10	6.1736	Down	chr9	–	Exonic	Kdelc1
rno_circRNA_0002969	4.17E-08	5.633	Down	chr13	–	Exonic	Cfh
rno_circRNA_0006911	1.80E-07	5.4753	Down	chr19	+	Exonic	Spg7
rno_circRNA_0006284	3.86E-07	5.3752	Down	chr18	+	Exonic	Wdr7
rno_circRNA_0001637	5.32E-07	5.3332	Down	chr11	–	Intronic	Ccdc80
rno_circRNA_0010322	1.05E-06	5.2562	Down	chr2	–	Exonic	Npnt
rno_circRNA_0014612	2.86E-06	5.1309	Down	chr5	+	Exonic	Rgs3
rno_circRNA_0012056	5.79E-10	5.064	Down	chr3	+	Intergenic	–
rno_circRNA_0015320	4.61E-06	5.0522	Down	chr6	–	Exonic	Hadhb
rno_circRNA_0019038	1.02E-18	4.8343	Down	chrX	+	Intergenic	–
rno_circRNA_0001500	5.59E-05	4.7371	Down	chr11	+	Intronic	Ttc3
rno_circRNA_0019050	0.000147	4.5686	Down	chrX	–	Intergenic	–
rno_circRNA_0000840	0.000236	4.481	Down	chr10	–	Exonic	Rph3al
rno_circRNA_0009286	0.000223	4.4693	Down	chr20	–	Exonic	Psmb9
rno_circRNA_0008598	0.000325	4.4257	Down	chr1	–	Exonic	AABR07007032.1
rno_circRNA_0007580	0.000423	4.3558	Down	chr1	+	Exonic	Pde3b
rno_circRNA_0009197	4.79E-08	4.1701	Down	chr20	–	Exonic	Cdh23
rno_circRNA_0011093	0.001512	4.1075	Down	chr3	+	Exonic	Slc27a2
rno_circRNA_0017128	0.001937	4.0647	Down	chr8	+	Exonic	Snrk

*circRNAs, circular RNAs; SHR, spontaneously hypertensive rats; Chrom, chromosome; Adcy5, adenylate cyclase 5; Fbn1, fibrillin-1; Cfh, complement factor H*.

### Prediction of circRNA-miRNA Interactions

**Figure 4:**
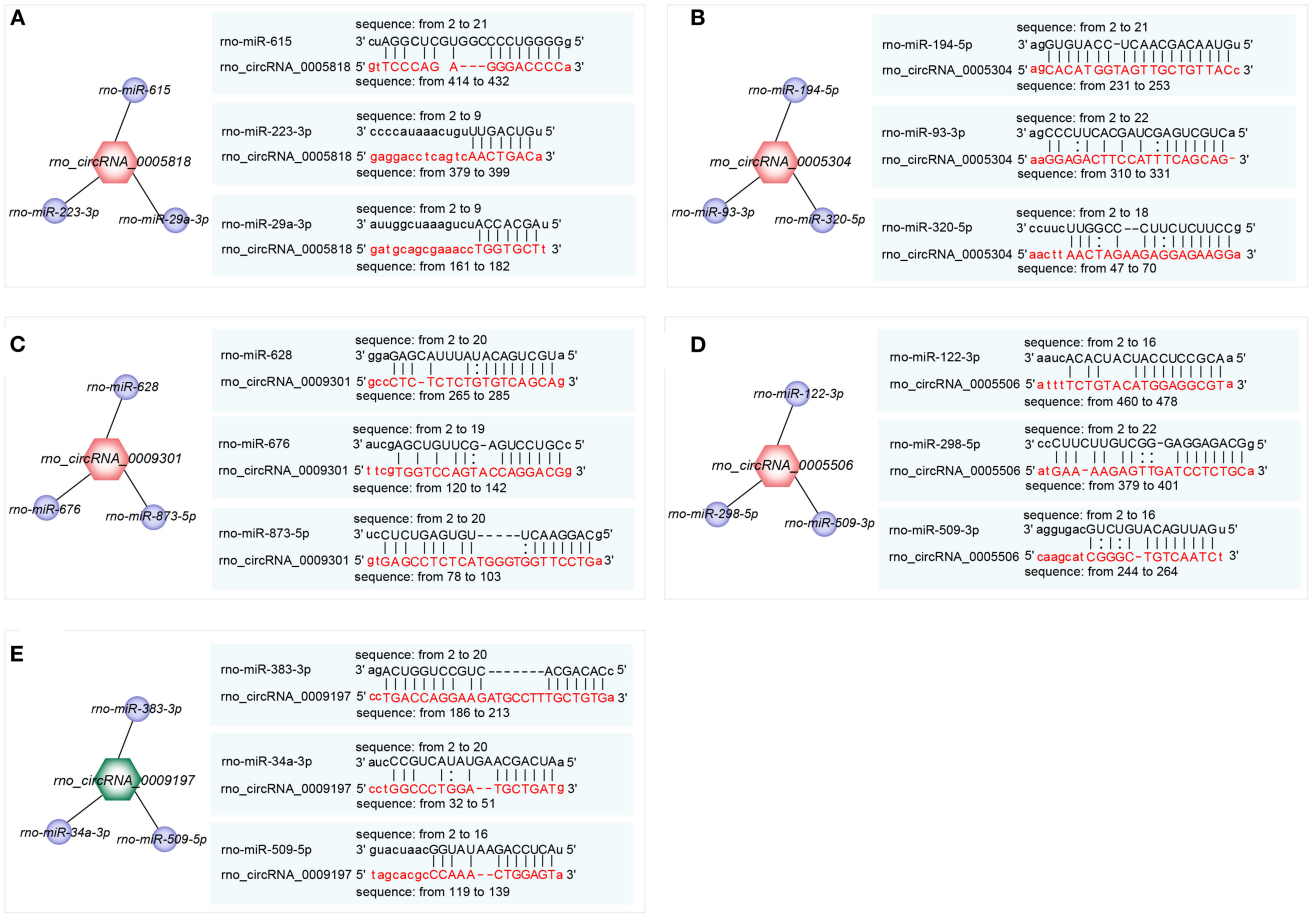
Prediction of the binding sites of circRNA-miRNA. **(A–E)** The top 3 miRNAs associated with each selected circRNA and the predicted binding sites. **(A)** rno_ circRNA_0005818 target pairs: rno_circRNA_0005818→ rno-miR-615; rno_circRNA_0005818→ rno-miR-223-3p; rno_circRNA_0005818→ rno-miR-29a-3p; **(B)** rno_circRNA_0005304 target pairs: rno_circRNA_0005304→ rno-miR-194-5p; rno_circRNA_0005304→ rno-miR-93-3p; rno_circRNA_0005304→ rno-miR-320-5p; **(C)** rno_circRNA_0009301 target pairs: rno_circRNA_0009301→ rno-miR-628; rno_circRNA_0009301→ rno-miR-676; rno_circRNA_0009301→ rno-miR-873-5p; **(D)** rno_circRNA_0005506 target pairs: rno_circRNA_0005506→ rno-miR-122-3p; rno_circRNA_0005506→ rno-miR-298-5p; rno_circRNA_0005506→ rno-miR-509-3p; **(E)** rno_circRNA_0009197 target pairs: rno_circRNA_0009197→ rno-miR-383-3p; rno_circRNA_0009197→ rno-miR-34a-3p; rno_circRNA_0009197→ rno-miR-509-5p. Target miRNAs of confirmed circRNAs were predicted using the miRanda software (http://www.miranda.org). circRNA, circular RNA; miRNA, microRNA.

The top 3 target miRNAs of confirmed circRNAs were predicted by using the miRanda software. Notably, rno-miR-615, rno-miR-223-3p, and rno-miR-29a-3p were predicted to construct ceRNA relationships with rno_circRNA_0005818 (Figure 4A). Rno-miR-194-5p, rno-miR-93-3p, and rno-miR-320-5p were predicted to construct ceRNA relationships with rno_circRNA_0005304 (Figure 4B). Moreover, rno-miR-628, rno-miR-676, and rno-miR-873-5p were predicted to construct ceRNA relationships with rno_circRNA_0009301 (Figure 4C). Rno-miR-122-3p, rno-miR-298-5p, and rno-miR-509-3p were predicted to build ceRNA relationships with rno_circRNA_0005506 (Figure 4D). Additionally, rno-miR-383-3p, rno-miR-34a-3p, and rno-miR-509-5p were predicted to construct ceRNA relationships with rno_circRNA_0009197 (Figure 4E). Collectively, circRNAs serve as a miRNA sponge associating with relevant miRNAs, which may further construct the circRNA-miRNA axis participating in pathogenesis of hypertension.

### Establishment of Validated circRNA-Related ceRNA Network in Aortic Vascular Tissues of Hypertensive Rats

**Figure 5:**
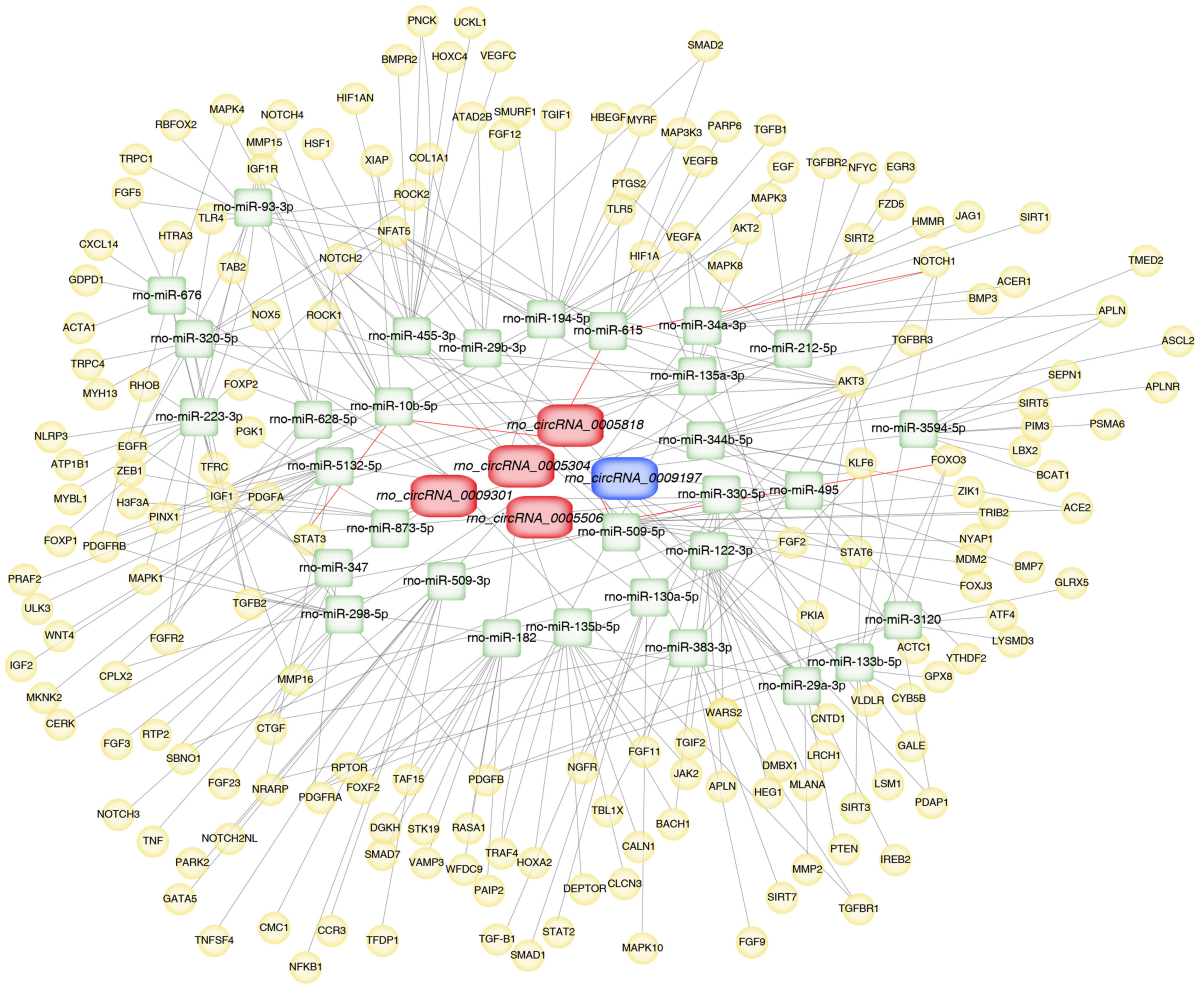
Construction of circRNA-miRNA-mRNA network in aortic vascular tissues of rats. The red color and blue color represent upregulated and downregulated significant DE circRNAs, respectively. Green color represents miRNAs, yellow color represents mRNAs. Red bold lines represent three promising circRNA-miRNA-mRNA regulatory axes were selected in hypertensive rat aorta, including rno_circRNA_0005818/miR-615/NOTCH1, rno_circRNA_0009197/miR-509-5p/FOXO3 and rno_circRNA_0005818/miR-10b-5p/STAT3, respectively. circRNAs, circular RNAs; miRNA, microRNA; mRNA, messenger RNAs; DE, differentially expressed.

To further explore the underlying mechanisms, the five validated circRNAs with related miRNAs as well as the downstream mRNAs were used to construct circRNA-miRNA-mRNA network using Cytoscape 3.8.2 software. The network was established with 31 predicted miRNAs and 266 mRNAs, which suggesting that circRNAs could modulate target miRNA indirectly by competing for miRNA-binding through common miRNA binding sites. Moreover, NOTCH1, a target of miR-615, was predicted to possess a ceRNA network with rno_circRNA_0005818. Forkhead box class O3 (FOXO3), a target of miR-509-5p, was predicted to possess a ceRNA network with rno_circRNA_0009197. Additionally, STAT3, a target of miR-10b-5p, was predicted to possess a ceRNA relationship with rno_circRNA_0005818 (Figure 5).

### Construction of PPI Network and Identification of Hub Genes in Rat Aorta

**Figure 6:**
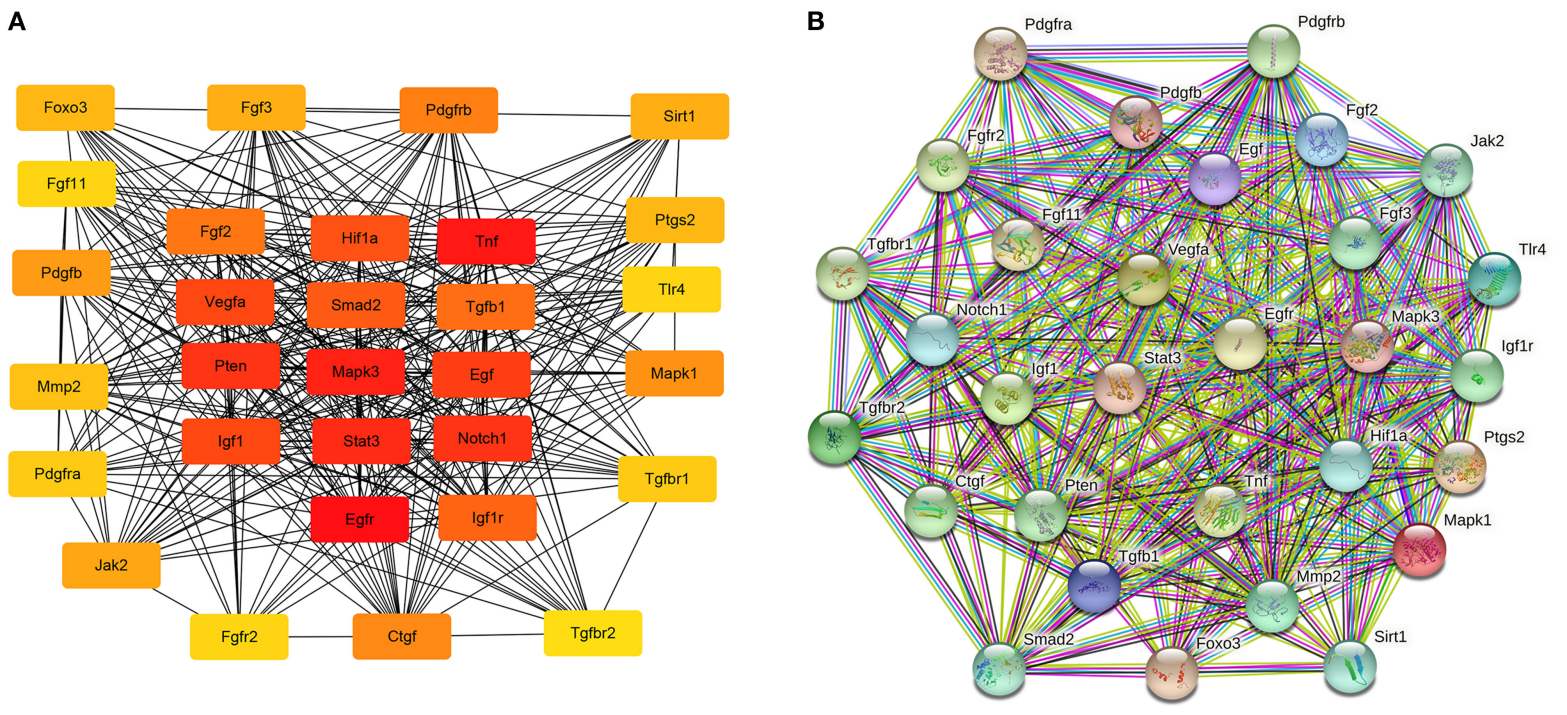
The potential interactions of hub genes in the PPI network in aortic vascular tissues of rats. **(A)** The top 30 hub genes were selected by the cytoHubba app in Cytoscape 3.8.2 software; The node color changes gradually from yellow to red in ascending order according to the score of hub genes. **(B)** The protein and protein interaction of top 30 hub genes by the online database STRING (involving 30 nodes and 344 edges). PPI, protein and protein interaction.

**Figure 7:**
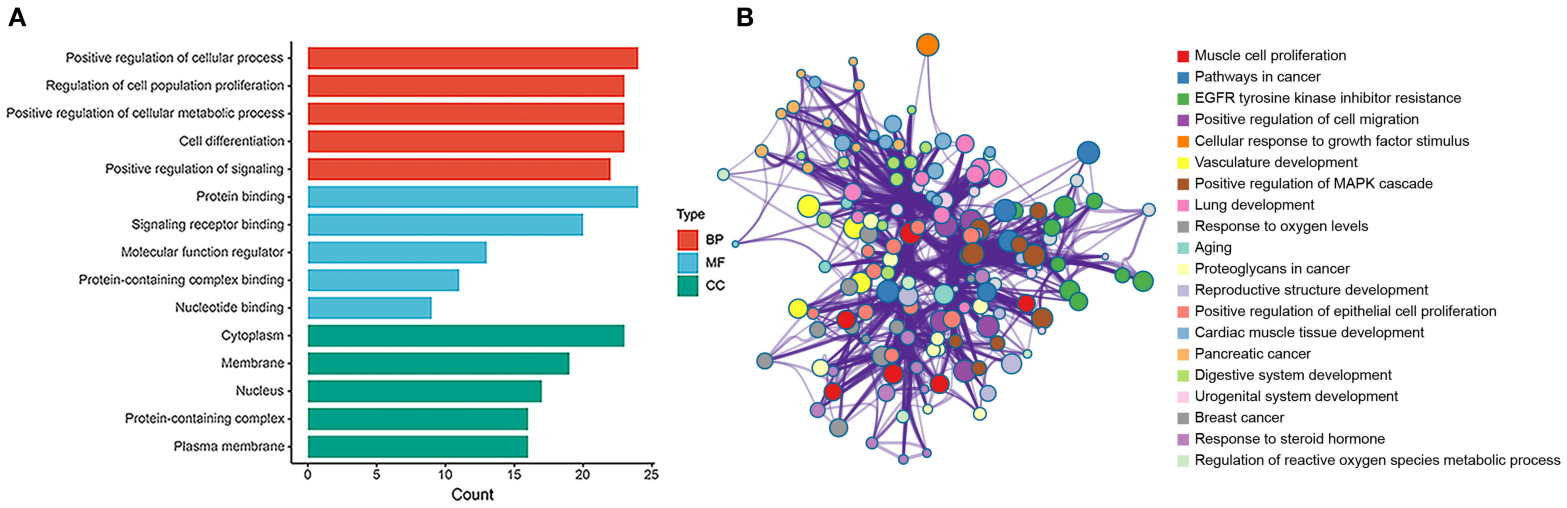
GO and enrichment network of top 30 hub genes between SHR and WKY rat aorta. **(A)** Top 5 GO enriched terms were analyzed in rat aorta for biological process (BP), cellular component (CC), and molecular function (MF), respectively. **(B)** The enrichment network of top 30 hub genes was identified in rat aorta. Nodes represent functions enriched for an annotated ontology term and node size indicates the number of genes that fall into that term. GO, gene ontology; WKY, Wistar-Kyoto; SHR spontaneously hypertensive rat; EGFR, epidermal growth factor receptor; MAPK, mitogen-activated protein kinase.

The hub genes were selected by the cytoHubba app in Cytoscape 3.8.2 software. Top 30 hub genes were identified in rat aorta, including signal transducer and activator of transcription 3 (STAT3), NOTCH1, FOXO3 (Figure 6A). Then, according to the STRING database, a PPI network (involving 30 nodes and 344 edges) was constructed (Figure 6B). The enriched GO terms of top 30 hub genes were conducted. Moreover, the top enriched GO-BP terms, included regulation of cell population proliferation and cell differentiation. There were several enriched terms related to GO-MF, such as protein binding, signaling receptor binding, molecular function regulator. In addition, GO-CC terms included cytoplasm, membrane and nucleus (Figure 7A). Furthermore, the top 20 GO and KEGG pathway of 30 hub genes were estimated by Metascape (Figure 7B), including epidermal growth factor receptor tyrosine kinase inhibitor resistance, vasculature development, regulation of cell migration and positive regulation of mitogen-activated protein kinases cascade.

## Discussion

CircRNAs form covalently closed continuous loops with high tissue specific expression, which are critical contributors to vascular pathophysiology and homeostasis (8, 9). CircRNAs are highly abundant in cardiovascular tissues, the regulation of circRNAs could mediate importantly physiological and pathological processes in the development of hypertension and related vascular diseases (9). In the present study, 485 DE circRNAs were found in aortic vascular tissues of hypertensive rats with 279 up-regulated circRNAs and 206 down-regulated circRNAs. Moreover, among these DE circRNAs, abundant RNAs were generated most commonly from exons of protein-coding genes (86.19 and 81.71% from up-regulated circRNAs and down-regulated circRNAs, respectively). Up till now, the understanding of circRNAs predicted functions in hypertensive rats remains largely unknown. To fully understand the functions of the confirmed circRNAs, clustering of GO terms performed that these circRNAs were enriched in the cytoplasmic part and GTPase regulator activity in molecular function. Besides, these circRNAs involved in the process of Endocytosis, Focal adhesion, cAMP signaling pathway, AMPK signaling pathway and ECM-receptor interaction by KEGG analysis. Therefore, based on bioinformatic analysis, we deduced that the significant confirmed circRNAs could link with hypertension and hypertensive vascular injury by modulating these signaling pathways.

Intriguingly, we further verified that downregulation of rno_circRNA_0009197, and upregulation of rno_circRNA_0005818, rno_circRNA_0005304, rno_circRNA_0005506, and rno_circRNA_0009301 were shown in aorta of SHR when compared with that of WKY rats by real-time RT-PCR analysis. These changes were associated with aggravated elastin and collagen fiber of thoracic aorta of hypertensive rats, implying that abnormal expression of aortic circRNAs may play potential roles in hypertensive vascular remodeling and dysfunction.

According to the circRNA-miRNA interactions, the circRNAs had been proven to combine with miRNAs binding sites and modulate the expression of mRNA through integrating with miRNA binding (9, 12). In the light of miRNA-mRNA interactions, a growing body of evidence indicated that the effects of miRNA were exerted by the target mRNAs (12, 13). In this work, circRNA-target miRNAs and the target mRNAs of miRNAs were predicted by the miRanda and Targetscan softwares, respectively. Then, the circRNA-miRNA-mRNA network was constructed, which provided a novel insight into the potential regulatory actions in the hypertension and hypertensive vascular injury. In accordance with previous reports, multiple miRNAs had been showed to modulate major genes participated in the development of hypertensive vascular injury. Subsequently, bioinformatics databases were used to predict the miRNAs that contained concrete and highly conserved binding sites of these significant confirmed circRNAs. We next to investigate the potential mainly regulator of miRNAs in vascular damage. To the best of our knowledge, fibrosis, oxidative stress, and ischemia have a close association with hypertension and ischemic heart diseases, which are the major pathways to cardiovascular remodeling (14). In response to the downstream miRNAs of the confirmed circRNAs, we revealed that miR-509-5p may be a promising target by inhibiting proliferation, migration and apoptosis in vasculature (15, 16). Vascular dysfunction is closely linked with endothelial cell (EC) dysfunction through abnormal gene modulation. The recent studies suggest that abnormal expression of miR-615 significantly repressed EC proliferation, migration, network tube formation in matrigel, the release of nitric oxide (17). Overexpression of miR-615 could reduce oxidative stress, apoptosis and ischemia involving in regulation of EC dysfunction and endothelial nitric oxide synthase (17, 18). Further, miR-10b-5p has vital actions on vascular remodeling (19), and has markedly less microscopic and macroscopic calcification nodules (20). At the same time, miR-10b-5p represses the myocardial fibrosis and ameliorated cardiomyocyte apoptosis and cardiac function after ischemia (21). However, different target genes of miRNAs have important biological functions in regulating target genes. Therefore, it is central to pay close attention on the underlying mechanisms of mRNAs in vascular pathophysiology in hypertension.

Combined with the PPI network, we found that three key hub genes played important roles in the PPI network. The three hub genes, STAT3, NOTCH1, and FOXO3, which are crucial essential factors to regulate the vascular diseases, and closely associated with modulation of proliferation, migration, differentiation and ischemia in a wide range of vascular diseases (22–24). STAT3, as a critical inducer and proangiogenic key regulator, is involved in EC proliferation, migration, and degradation of the ECM (25). STAT3 has been demonstrated to influence the expression of angiogenic and angiostatic mediators, such as basic fibroblast growth factor, vascular endothelial growth factor (VEGF) (26, 27). Additionally, STAT3 signaling might activate as a promising approach to inhibit the progression of vascular narrowing (28). The alternative splice variant of VEGF (VEGFxxxb) VEGF_165_b, modulates endothelial VEGF receptor 1-STAT3 signaling pathway in ischemia and peripheral arterial disease (29). NOTCH activation is a major pathogenic mechanism participated in the progression of pulmonary vascular remodeling (30). Repression of NOTCH1 could alleviate vascular lesion, including endothelial function-related factors, oxidative stress-related factors, and reduce apoptosis of aortic EC of hypertensive rats (31, 32). Interesting, NOTCH1 is necessary for VEGF-induced migration, proliferation and survival of EC (33). Activated Notch signaling and inhibited TGF-β1/Smad3 signaling could repress myocardial fibrosis after myocardial infarction (MI) (29). FOXO3, as a transcriptional factor and important mediator, has been shown to contribute to the protective effects of cardiovascular fibrosis and ischemia-reperfusion (I/R) injury (34). miR-629 promoted cell proliferation, migration and apoptosis by targeting FOXO3 in vascular remodeling (35). The functions of miR-124 on hypertensive pulmonary fibroblast proliferation were mediated through FOXO3/cdk inhibitor cdkn1a signaling (36). These results indicate that hub-genes play critical roles in proliferation,cell differentiation, ischemia, and cardiovascular fibrosis. In the present work, we selected three promising circRNA-miRNA-mRNA regulatory axes in hypertensive rat aorta, including rno_circRNA_0005818/miR-615/NOTCH1, rno_circRNA_0009197/miR-509-5p/FOXO3, rno_circRNA_0005818/miR-10b-5p/STAT3, which may have further exploration for the pathological process of hypertensive vascular injury and dysfunction.

In summary, we demonstrated, for the first time, new insight into the 485 DE circRNAs in SHR aorta compared with WKY rat aorta through RNA-seq array and real-time RT-PCR validation. Of these, 5 DE circRNAs were verified in hypertensive rat aorta. Particularly, rno_circRNA_0009197 was down-regulated in aortic vascular tissues of hypertensive rats with up-regulated levels of rno_circRNA_0005818, rno_circRNA_ 0005304, rno_circRNA_ 0005506, and rno_circRNA_0009301. More importantly, we found that there were highly similar homologous sequences in rno_circRNA_0009197 and rno_circRNA_0005506 with hsa_circ_0018685|chr10:73337670-73559386 and hsa_circ_0112669|chr1: 237865277-237921076, respectively. Moreover, three hub genes (NOTCH1, FOXO3, and STAT3) according to PPI network were found. Furthermore, based on the ceRNA regulatory mechanism, the circRNA-miRNA-mRNA network was constructed for three promising circRNA-miRNA-mRNA regulatory axes, including rno_circRNA_0005818/miR-615/NOTCH1, rno_circRNA_0009197/miR-509-5p/FOXO3, and rno_circRNA_0005818/miR-10b-5p/STAT3, respectively. Therefore, our findings exhibited that aortic circRNAs play potential roles in regulating hypertensive vascular remodeling and dysfunction and aortic circRNAs are the vital therapeutic targets for hypertension-related vascular diseases.

## Data Availability Statement

The datasets presented in this study can be found in online repositories. The names of the repository/repositories and accession number(s) can be found at: NCBI SRA; PRJNA785011.

## Ethics Statement

The animal study was reviewed and approved by National Institutes of Health guide for the care and use of Laboratory animals (NIH Publications No. 8023). Animal Research Ethics Committee of Beijing Chaoyang Hospital affiliated to Capital Medical University.

## Author Contributions

YL, YD, and ZD: writing-original draft, methodology, supervision, writing-review and editing, read, and approved the final manuscript. JS, ZZ, LL, and XL: collected and recorded the samples, read, and approved the final manuscript. LS, XL, MZ, and YC: formal analysis, read, and approved the final manuscript. JZ and RM: methodology, supervision, writing—review and editing, read, and approved the final manuscript. All authors contributed to the article and approved the submitted version.

## Funding

This study was supported by the General Program and the National Major Research Plan Training Program of the National Natural Science Foundation of China (Nos. 81770253, 91849111, 81370362, 82170302, and 81300044), Shanghai Sailing Program (20YF1444100), Beijing Natural Science Foundation (7162069), Beijing Hospitals Authority Youth Programme (QML20200305), Open Foundation from Beijing Key Laboratory of Hypertension Research (2019GXY-KFKT-02), Talent Project of Beijing Chaoyang Hospital Affiliated to Capital Medical University.

## Conflict of Interest

The authors declare that the research was conducted in the absence of any commercial or financial relationships that could be construed as a potential conflict of interest.

## Publisher's Note

All claims expressed in this article are solely those of the authors and do not necessarily represent those of their affiliated organizations, or those of the publisher, the editors and the reviewers. Any product that may be evaluated in this article, or claim that may be made by its manufacturer, is not guaranteed or endorsed by the publisher.
