# Supplementary material for: Expression Profiles of Circular RNA in Aortic Vascular Tissues of Spontaneously Hypertensive Rats
Source: Front Cardiovasc Med. 2021 Dec 20;8:814402. doi: 10.3389/fcvm.2021.814402 (PMC8720857; doi:10.3389/fcvm.2021.814402)
Supplement: Supplementary file 1 [file Data_Sheet_1.doc]

**Expression Profiles of Circular RNA in** **Aortic Vascular Tissues of Spontaneously Hypertensive Rats**

**Ying Liu^†^, Ying Dong^†^, Zhaojie Dong^†^, Jiawei Song, Zhenzhou Zhang, Lirong Liang, Xiaoyan Liu, Lanlan Sun, Xueting Li, Miwen Zhang, Yihang Chen, Ran Miao^*^, Jiuchang Zhong^*^**

**^†^** These authors contributed equally to this work.

***Corresponding author:** Jiuchang Zhong, MD or Ran Miao MD, Heart Center and Beijing Key Laboratory of Hypertension, Beijing Institute of Respiratory Medicine and Beijing Chaoyang Hospital, Capital Medical University, Beijing 100020, China; Email: [jczhong@sina.com](mailto:jczhong@sina.com) or [mr1019@163.com](mailto:Mr1019@163.com) Phone: (+86)-10-85231939; Fax: (+86)-10-85231939. **ORCID:** Jiuchang Zhong: <https://orcid.org/0000-0002-2315-3515>.

**SUPPLEMENTARY MATERIAL**

**Supplementary Figure and Tables**


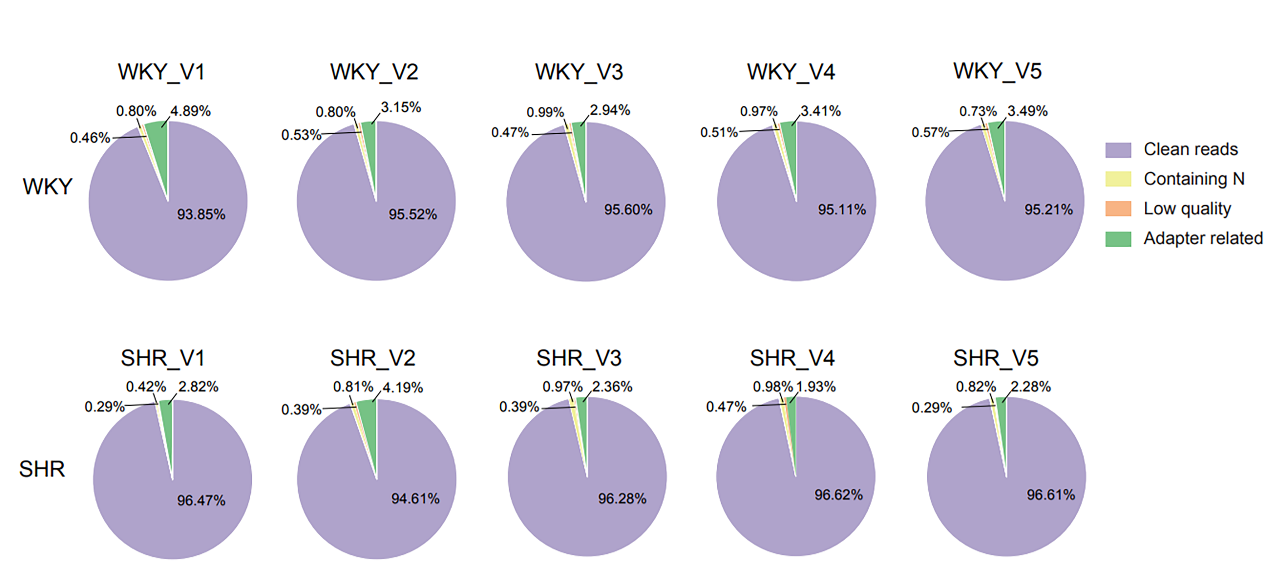


**Figure S1.** The nucleic acid sequence and sequencing quality for circular RNA in rat aorta. The classification of the nucleic acid sequence and sequencing quality for circular RNA in SHR and WKY rats, including clean reads, containing N, low quality, and adapter related.

WKY: Wistar-Kyoto; SHR, spontaneously hypertensive rats.

**Table S1.** Information of raw data for circular RNA of aorta in SHR and WKY rats.

| **Sample** | **Raw reads** | **Clean reads** | **Raw bases (G)** | **Clean bases(G)** | **Error rate (%)** | **Q20 (%)** | **Q30**  **(%)** | **GC content (%)** |
| --- | --- | --- | --- | --- | --- | --- | --- | --- |
| SHR_V1 | 73517532 | 70922280 | 11.03 | 10.64 | 0.02 | 98.37 | 95.29 | 57.04 |
| SHR_V2 | 80323532 | 75991564 | 12.05 | 11.4 | 0.03 | 97.87 | 94.08 | 56.06 |
| SHR_V3 | 75289162 | 72485208 | 11.29 | 10.87 | 0.03 | 97.84 | 93.94 | 55.11 |
| SHR_V4 | 69982302 | 67609710 | 10.5 | 10.14 | 0.03 | 97.68 | 93.64 | 55.61 |
| SHR_V5 | 79975118 | 77256742 | 12 | 11.59 | 0.02 | 98.02 | 94.34 | 56.85 |
| WKY_V1 | 79400008 | 74519846 | 11.91 | 11.18 | 0.03 | 97.9 | 94.23 | 57.22 |
| WKY_V2 | 76880304 | 73442826 | 11.53 | 11.02 | 0.03 | 97.55 | 93.38 | 56.87 |
| WKY_V3 | 70100004 | 67018090 | 10.52 | 10.05 | 0.03 | 97.75 | 93.84 | 57.08 |
| WKY_V4 | 74911454 | 71250882 | 11.24 | 10.69 | 0.03 | 97.72 | 93.82 | 57.17 |
| WKY_V5 | 78720802 | 74953776 | 11.81 | 11.24 | 0.03 | 97.8 | 94.06 | 57.04 |

WKY: Wistar-Kyoto; SHR, spontaneously hypertensive rats.

**Table S2.** Primers designed for real-time PCR (RT-PCR) validation of altered circRNAs.

| **Gene name** | **Forward and reverse primer (5`-3`)** | **Tm (℃)** | **Product length (bp)** |
| --- | --- | --- | --- |
| rno_circRNA_0005818 | Forward-ACCTGAATGGACAGAAGAGGA  Reverse-TTCTCACTCTCCTGTAGTAGAA | 60 | 189 |
| rno_circRNA_0009197 | Forward-GGAGTATCACATCCTTAGCAT  Reverse-ATAACAAACAGGTCGCTGTGA | 60 | 204 |
| rno_circRNA_0002616 | Forward-ATTGGTTGAAGAACTCCATCC  Reverse-ATTATCTCTGGAGTGGATGCTC | 60 | 205 |
| rno_circRNA_0005304 | Forward-AGTAGCACATGGTAGTTGCTG  Reverse-TCTTCTCCTTCTCCTCTTCTAG | 60 | 179 |
| rno_circRNA_0005506 | Forward-GTTGTCACGATGAAGAAGACG  Reverse-AAGCACATTCGCTATATCCAG | 60 | 173 |
| rno_circRNA_0006911 | Forward-AGATGATGATAAACCCAAAGGC  Reverse-ATGCCGTTTCAGTAACAATCCG | 60 | 110 |
| rno_circRNA_0009301 | Forward-AGATGAACCTGTATGGATTCCATG  Reverse-AGTCACTGCCAACTCCTCCAG | 60 | 125 |
| Rat GAPDH | Forward- AGTGCCAGCCTCGTCTCATA  Reverse- TGAACTTGCCGTGGGTAGAG | 60 | 197 |

Tm: temperature; Bp, base pair; circRNAs, circular RNAs; GAPDH, Glyceraldehyde-3 phosp

hate dehydrogenase; RT-PCR, real-time polymerase chain reaction.

**Table S3.** Result of KEGG pathway analysis of circRNAs related to hypertension.

| **Pathway term** | **Rich factor** | **Q value** | **Gene number** |
| --- | --- | --- | --- |
| Phosphatidylinositol signaling system | 0.1111 | 0.0718 | 9 |
| Endocytosis | 0.0612 | 0.2026 | 15 |
| Adipocytokine signaling pathway | 0.0946 | 0.2421 | 7 |
| Inositol phosphate metabolism | 0.0909 | 0.4075 | 6 |
| Proteoglycans in cancer | 0.0545 | 0.4964 | 11 |
| PPAR signaling pathway | 0.0759 | 0.4964 | 6 |
| Focal adhesion | 0.0529 | 0.4964 | 11 |
| Dopaminergic synapse | 0.0611 | 0.4964 | 8 |
| Cholinergic synapse | 0.0631 | 0.4964 | 7 |
| cAMP signaling pathway | 0.0513 | 0.4964 | 10 |
| GnRH signaling pathway | 0.0667 | 0.4964 | 6 |
| Renal cell carcinoma | 0.0735 | 0.4964 | 5 |
| cGMP-PKG signaling pathway | 0.0526 | 0.4964 | 9 |
| Adrenergic signaling in cardiomyocytes | 0.0541 | 0.4964 | 8 |
| Mucin type O-Glycan biosynthesis | 0.1071 | 0.4964 | 3 |
| Estrogen signaling pathway | 0.0619 | 0.4964 | 6 |
| Endometrial cancer | 0.0769 | 0.5427 | 4 |
| AMPK signaling pathway | 0.0538 | 0.5589 | 7 |
| Fatty acid biosynthesis | 0.1429 | 0.5719 | 2 |
| ECM-receptor interaction | 0.0581 | 0.6662 | 5 |

KEGG: Kyoto encyclopedia of genes and genomes; circRNAs, circular RNAs; PPAR, peroxisome proliferator activated receptor; cAMP, cyclic adenosine monophosphate; GnRH, gonadotropin-releasing hormone; cGMP, cyclic guanosine monophosphate; PKG, protein kinase G; AMPK, adenosine 5‘-monophosphate-activated protein kinase; ECM, extracellular matrix.

**Table S4.** Result of GO terms analysis of circRNAs related to hypertension.

| **GO Term** | **Category** | **Description** | **P value** |
| --- | --- | --- | --- |
| GO:0046361 | BP | 2-oxobutyrate metabolic process | 3.79E-05 |
| GO:0048268 | BP | clathrin coat assembly | 5.71E-05 |
| GO:0010603 | BP | regulation of cytoplasmic mRNA processing body assembly | 6.76E-05 |
| GO:0006629 | BP | lipid metabolic process | 7.33E-05 |
| GO:0007507 | BP | heart development | 1.67E-5 |
| GO:0046486 | BP | glycerolipid metabolic process | 0.2E-4 |
| GO:2000403 | BP | positive regulation of lymphocyte migration | 0.22E-4 |
| GO:0044710 | BP | single-organism metabolic process | 2.72E-5 |
| GO:0097089 | BP | methyl-branched fatty acid metabolic process | 2.73E-5 |
| GO:0007044 | BP | cell-substrate junction assembly | 2.87E-5 |
| GO:0044444 | CC | cytoplasmic part | 2.60E-08 |
| GO:0005737 | CC | cytoplasm | 1.33E-07 |
| GO:0044424 | CC | intracellular part | 7.54E-07 |
| GO:0043229 | CC | intracellular organelle | 8.84E-07 |
| GO:0043226 | CC | organelle | 1.42E-06 |
| GO:0005622 | CC | intracellular | 1.64E-06 |
| GO:0043231 | CC | intracellular membrane-bounded organelle | 2.07E-5 |
| GO:0044422 | CC | organelle part | 2.58E-5 |
| GO:0043227 | CC | membrane-bounded organelle | 2.72E-5 |
| GO:0044446 | CC | intracellular organelle part | 6.35E-5 |
| GO:0030695 | MF | GTPase regulator activity | 1.44E-05 |
| GO:0060589 | MF | nucleoside-triphosphatase regulator activity | 2.91E-05 |
| GO:0030234 | MF | enzyme regulator activity | 3.33E-05 |
| GO:0035091 | MF | phosphatidylinositol binding | 3.96E-05 |
| GO:0005515 | MF | protein binding | 5.57E-05 |
| GO:0005488 | MF | binding | 2.7E-5 |
| GO:0017137 | MF | Rab GTPase binding | 4.5E-5 |
| GO:0019899 | MF | enzyme binding | 7.04E-5 |
| GO:0016174 | MF | NAD(P)H oxidase activity | 1.169E-4 |
| GO:0005083 | MF | small GTPase regulator activity | 1.61E-4 |

CircRNAs: circular RNAs; GO, gene ontology; BP, biological process; CC, cellular component; MF, molecular function; GTPase, Guanosine-Triphosphate hydrolase; NADH, Nicotinamide adenine dinucleotide; NADPH, nicotinamide adenine dinucleotide phosphate.
